# Processed Diets and Food Additives Shape the Gut Microbiota and Chronic Disease Risk Across the Life Course—A Three-Layer Ecosystem Disruption Model (TLED) Model

**DOI:** 10.3390/life16030505

**Published:** 2026-03-19

**Authors:** Monica Manciulea (Profir), Luciana Alexandra Pavelescu, Gabriel Florin Răzvan Mogoş, Alin Constantin Stancu, Sanda Maria Cretoiu, Ileana Marinescu

**Affiliations:** 1 Department of Morphological Sciences, Cell and Molecular Biology and Histology, Carol Davila University of Medicine and Pharmacy, 050474 Bucharest, Romania; monica.profir@rez.umfcd.ro (M.M.); luciana.pavelescu@umfcd.ro (L.A.P.); alin-constantin.stancu@rez.umfcd.ro (A.C.S.); 2 Department of Surgery, University of Medicine and Pharmacy of Craiova, 200349 Craiova, Romania; gabrielmogos@yahoo.com; 3 Department of Psychiatry, Faculty of Medicine, University of Medicine and Pharmacy of Craiova, 200349 Craiova, Romania; ileana.marinescu@umfcv.ro; 4 Psychiatry Clinic, Craiova Clinical Hospital for Neuropsychiatry, 200615 Craiova, Romania

**Keywords:** ultra-processed foods, food additives, multi-additive exposure, gut microbiota, ecosystem resilience, intestinal barrier, short-chain fatty acids, immune reprogramming, life-course, dysbiosis, microbiological scarring, chronic inflammation

## Abstract

Ultra-processed foods (UPFs) represent a distinct dietary paradigm characterized by structurally simplified food matrices and chronic exposure to multiple additives, including emulsifiers, artificial sweeteners, and preservatives. Rather than acting in isolation, these compounds operate within a multi-additive environment that reshapes the gut ecosystem through convergent mechanisms. Emerging evidence suggests that additive-rich ultra-processed dietary environments may disrupt the gut ecosystem through three interconnected layers: (1) structural impairment of the intestinal barrier, including mucus erosion and tight-junction destabilization; (2) microbial metabolic shifts marked by short-chain fatty acid depletion, altered bile acid signaling, and enrichment of lipopolysaccharide-producing taxa; and (3) immune and inflammatory reprogramming promoting low-grade systemic inflammation. These processes collectively reduce ecosystem resilience—the capacity of the gut microbiota to resist and recover from perturbation. Vulnerability to additive-driven dysbiosis varies across the life course. During infancy, incomplete ecosystem stabilization may increase susceptibility to long-term ecological imprinting, whereas in older age, reduced microbial diversity and immune remodeling may impair recovery capacity following dietary stressors. In contrast, fiber-rich, minimally processed dietary patterns appear to enhance microbial resilience by reinforcing functional redundancy, metabolic buffering, and barrier integrity. Although much mechanistic evidence has been derived from experimental models, accumulating human data support the biological plausibility of additive-associated microbiota alterations. By integrating multi-additive exposure, ecosystem disruption, life-course modulation, and resilience within a unified framework, this review provides a mechanistically coherent model linking ultra-processed dietary environments to microbiota-mediated chronic disease risk. Here, we formalize this integrative perspective as the Three-Layer Ecosystem Disruption (TLED) Model.

## 1. Introduction

The concept of diet, derived from the Greek word *díaita* (“way of life”), reflects the deep interconnection between dietary habits, lifestyle, and health. Dietary patterns are shaped by geographical, cultural, social, and economic factors and exert profound effects on quality of life, disease risk, and longevity [1].

In recent decades, global dietary habits have undergone a marked transition, characterized not only by changes in macronutrient composition but by a fundamental structural transformation of the food matrix itself. This nutritional transition has coincided with a dramatic rise in non-communicable diseases (NCDs), including diabetes, cardiovascular disease, cancer, and chronic respiratory conditions, which now account for the majority of global mortality [2].

Diet represents a major modifiable risk factor underlying this burden, particularly the shift from traditional, minimally processed diets toward modern dietary patterns dominated by ultra-processed foods (UPFs) [3,4].

These foods are characterized by refined substrates, low dietary fiber content, altered physical structures, and the widespread use of synthetic additives such as emulsifiers, artificial sweeteners, preservatives, and colorants.

Increasing evidence indicates that UPFs represent a biologically distinct nutritional paradigm, with health effects that extend beyond caloric density or macronutrient ratios. A central mediator of these effects is the gut microbiota, a metabolically active microbial ecosystem increasingly recognized as a key regulator of host physiology. The gut microbiota plays a crucial role in nutrient metabolism, immune system development, maintenance of mucosal barrier integrity, metabolic signaling, and inflammatory regulation [5].

Diet is one of the strongest modulators of gut microbiota composition and function, capable of inducing rapid and reproducible changes in microbial communities [5].

A healthy gut microbiota, characterized by diversity, functional redundancy, and ecological stability (eubiosis), supports immune tolerance and metabolic homeostasis, whereas dysbiosis—marked by reduced diversity, altered community structure, and functional imbalance—has been linked to a wide range of chronic diseases, including obesity, type 2 diabetes (T2D), inflammatory bowel disease, cardiovascular disease, and cancer [5,6].

While the adult human gut microbiota is typically dominated by the phyla *Firmicutes* and *Bacteroidetes*, its composition remains dynamic and highly responsive to dietary, environmental, and lifestyle factors throughout life [7]. Diets rich in UPFs, low in fiber, and high in saturated fats, refined sugars, and food additives have been consistently associated with reduced microbial diversity, depletion of beneficial taxa, enrichment of pro-inflammatory or opportunistic species, impaired intestinal barrier integrity, and chronic low-grade inflammation [5].

Importantly, emerging evidence suggests that the effects of modern processed diets are not limited to isolated nutrients or single additives. Rather, chronic exposure to additive-rich UPFs induces cumulative, multi-layered disruptions of the gut ecosystem, affecting microbial composition, metabolic output, mucosal integrity, and host immune responses. These effects may help explain the strong epidemiological links between Western dietary patterns and metabolic dysfunction, inflammatory diseases, and colorectal cancer [5].

Crucially, susceptibility to diet-induced disruption of the microbiota is not uniform across the lifespan. Early life is characterized by high microbial plasticity and critical windows of immune and metabolic imprinting, rendering the developing microbiome particularly sensitive to dietary exposures. In adulthood, cumulative exposure to processed foods may progressively erode microbial resilience, while ageing is associated with reduced functional redundancy, immune senescence, and diminished capacity for recovery following perturbation. A life-course perspective, therefore, provides a more mechanistically coherent framework for understanding how processed dietary environments shape long-term disease risk.

Rather than providing a purely descriptive synthesis of additive-specific findings, this review proposes a conceptual framework integrating structural barrier disruption, microbial metabolic reprogramming, and immune activation within a life-course-dependent resilience model. By situating multi-additive exposure within a systems-level ecological perspective, we aim to reframe ultra-processed dietary environments as coordinated perturbation fields rather than isolated chemical effects.

## 2. Ultra-Processed Diets and Additive Exposure: Mechanistic Disruption of the Gut Ecosystem

The NOVA defines UPFs as industrially made items intended for convenience, enhanced palatability, and extended shelf life; they often include food-derived chemicals as well as cosmetic additives [8].

The modern Western dietary environment is characterized by sustained consumption of UPFs, which expose the gut ecosystem to chronic, low-dose mixtures of emulsifiers, artificial sweeteners, preservatives, colorants, and emerging environmental contaminants. Epidemiological data link additive-rich dietary patterns to colon cancer, inflammatory bowel disease, obesity, insulin resistance, and metabolic-associated fatty liver disease [9,10]. Importantly, real-world dietary exposure does not involve isolated compounds but rather continuous multi-additive co-exposure embedded within structurally altered, fiber-depleted food matrices. Rather than acting independently, these exposures converge on three interrelated pathogenic layers: structural disruption, metabolic reprogramming, and immune activation.

A growing body of mechanistic literature indicates that selected additive classes commonly found in UPFs—including emulsifiers, non-nutritive sweeteners, and antimicrobial preservatives—can perturb the gut ecosystem (microbiota composition/function, mucus barrier properties, epithelial integrity, and immune–metabolic signaling), particularly in experimental models [11,12,13]. Because real-world UPF exposure usually involves chronic, low-dose co-exposure to multiple additives, emerging mixture-focused epidemiology supports evaluating combined additive patterns as a distinct exposure paradigm relevant to metabolic disease risk.

**Figure 1:**
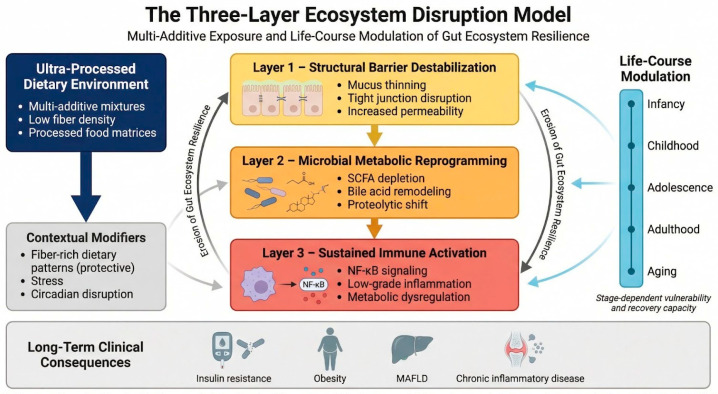
Three-Layer Model of Gut Ecosystem Disruption (Structural—Metabolic—Immune). This diagram illustrates a three-layer pathogenic cascade triggered by chronic exposure to ultra-processed dietary environments. Layer 1 involves structural barrier destabilization, characterized by mucus thinning, tight-junction disruption, and increased intestinal permeability. Layer 2 reflects microbial metabolic reprogramming, including short-chain fatty acid depletion, bile acid remodeling, and a shift toward proteolytic fermentation. Layer 3 represents sustained immune activation, marked by NF-κB signaling, low-grade inflammation, and metabolic dysregulation. The magnitude and reversibility of this cascade are modulated across the life course, ultimately contributing to long-term clinical outcomes such as insulin resistance, obesity, Metabolic Dysfunction-Associated Fatty Liver Disease (MAFLD), and chronic inflammatory disease. This figure was generated using the FigureLabs AI platform (https://chat.figurelabs.ai) based on the authors’ original concept and instructions; the final image was reviewed and edited by the authors.

We propose the Three-Layer Ecosystem Disruption Model (TLED Model) (Figure 1) in which chronic multi-additive exposure embedded within ultra-processed dietary matrices induces:

1. Structural barrier destabilization;
2. Microbial metabolic reprogramming;
3. Sustained immune activation.

These effects collectively lead to erosion of gut ecosystem resilience across the life course.

### 2.1. Additive-Driven Structural Dysfunction: Mucus Erosion and Tight-Junction Destabilization

The first mechanistic layer of ultra-processed food-associated gut disruption involves impairment of intestinal barrier architecture, including mucus integrity and epithelial tight-junction organization. In the colon, the mucus barrier normally limits direct microbial contact with host tissues; the inner mucus layer is dense and largely impenetrable to bacteria under homeostatic conditions, preserving epithelial sterility at the luminal interface. Goblet cells synthesize and secrete the gel-forming mucin MUC2, which is essential for mucus barrier integrity and mucosal immune homeostasis. Disruption of mucus production or altered mucus physicochemical properties increases susceptibility to inflammation by permitting closer microbe–epithelium interactions [14,15].

In preclinical models, common dietary emulsifiers such as carboxymethylcellulose (CMC) and polysorbate-80 (P80) have been shown to alter microbiota composition and promote features consistent with impaired mucus–epithelial spatial organization, including increased microbial encroachment toward the epithelium [9,11,16,17]. These structural alterations are accompanied by expansion of mucolytic and pro-inflammatory taxa—including *Proteobacteria* and *Ruminococcus gnavus*—which are capable of penetrating the mucus barrier, alongside depletion of protective organisms such as *Akkermansia muciniphila* and members of Bacteroidetes [18].

In murine systems, emulsifier exposure has been associated with low-grade inflammation, metabolic syndrome–like phenotypes, and exacerbation of colitis in susceptible models, supporting a microbiota- and barrier-linked mechanism [11]. More recent experimental syntheses continue to cite mucus thinning, increased epithelial permeability, and altered lipopolysaccharide (LPS)-related signaling as plausible downstream pathways in emulsifier-driven host responses, while emphasizing that strength of evidence varies across compounds, doses, and models [12,19].

It is important to acknowledge that much of the mechanistic evidence derives from murine models in which administered concentrations may exceed average human intake on a body-weight basis. Although several studies aim to approximate regulatory acceptable daily intake (ADI) levels, interspecies differences in microbiota composition, metabolism, and exposure patterns complicate direct extrapolation. Notably, a randomized controlled-feeding human study evaluating dietary CMC demonstrated measurable perturbations in gut microbiota composition and fecal metabolomic profiles during short-term exposure within ranges relevant to processed food consumption [20,21]

Within that trial, a subset of participants exhibited microbiota encroachment into the normally bacteria-sparse inner mucus layer, indicating inter-individual susceptibility to mucus–microbe spatial disruption under emulsifier/thickener exposure. Commentary accompanying these findings underscores the importance of evaluating ubiquitous additives under real-world dietary conditions and highlights biological heterogeneity in host response. Nevertheless, the extent to which chronic low-dose mixture exposure in humans recapitulates murine findings remains incompletely defined. Beyond emulsifiers, in vitro intestinal epithelial models demonstrate that artificial sweeteners such as sucralose, saccharin, and aspartame increase paracellular permeability through downregulation and mislocalization of tight-junction proteins, including claudin-3, occludin, and ZO-1 [22,23]. These effects are mediated, at least in part, by oxidative stress-dependent signaling and activation of NF-κB pathways. Saccharin exposure promotes NF-κB-mediated downregulation and degradation of claudin-1, directly compromising epithelial barrier integrity [13,24].

Additional structural stressors within ultra-processed dietary environments include certain food colorants, nanoparticulate additives, and emerging contaminants such as micro- and nanoplastics. Experimental studies suggest that these compounds can activate inflammasomes, alter mucosal immune responses, and increase intestinal permeability [25]. In murine models, polylactic acid (PLA) microplastics have been shown to induce gut dysbiosis, including expansion of Lachnospiraceae, and to contribute to hepatotoxicity [26].

Collectively, emulsifier-induced mucus erosion, tight-junction destabilization, additive-driven oxidative stress, and contaminant-associated epithelial injury converge to reduce barrier compartmentalization and facilitate translocation of microbial products into the lamina propria and systemic circulation. This structural destabilization constitutes the first pathogenic layer linking ultra-processed dietary exposure to downstream metabolic and immune dysregulation.

### 2.2. Metabolic Shift: SCFA Depletion, Bile Acid Reconfiguration, and Proteolytic Fermentation

The second pathogenic layer reflects functional reprogramming of microbial metabolic output under additive-rich dietary conditions. At the community level, emulsifier- and sweetener-rich environments suppress short-chain fatty acid (SCFA)-producing taxa, including butyrate-producing Firmicutes, while favoring Gram-negative, lipopolysaccharide-producing bacteria [11,16,17]. Reduced SCFA availability compromises colonocyte energy metabolism, mucin synthesis, and regulatory T-cell differentiation, thereby weakening epithelial immune tolerance and metabolic homeostasis [27,28,29].

Recent experimental evidence further demonstrates that CMC exposure promotes expansion of potentially pathogenic genera, including *Clostridium* and *Escherichia–Shigella*, while reducing beneficial taxa such as *Lactobacillus* and *Akkermansia* [30].

Artificial sweeteners exert parallel metabolic effects. Bian et al. reported sex-specific dysbiosis following acesulfame-K exposure in CD-1 mice, with enrichment of *Sutterella* and *Bacteroides* in males and depletion of *Clostridium* and *Lactobacillus* in females [31,32].

Prolonged sucralose intake has been associated with reduced luminal butyrate levels and enrichment of *Blautia* spp. and *Bacteroides*, indicating altered microbial metabolic signaling and substrate utilization [9,24].

Beyond SCFA depletion, additive-induced dysbiosis is characterized by altered bile acid metabolism and increased proteolytic fermentation, shifting microbial output toward secondary bile acids and other pro-inflammatory metabolites [16,30].

Such changes reflect not only taxonomic shifts, but also reconfigurations of host–microbe metabolic cross-talk, with downstream consequences for insulin sensitivity, lipid metabolism, and inflammatory tone.

### 2.3. Immune Reprogramming: Sustained Low-Grade Inflammatory Tone

Structural barrier compromise combined with enrichment of Gram-negative taxa enhances systemic exposure to lipopolysaccharides (LPSs), promoting metabolic endotoxemia. Elevated circulating LPS levels have been mechanistically linked to insulin resistance, adipose tissue inflammation, and chronic low-grade immune activation [16,33].

Experimental studies demonstrate that emulsifier exposure induces colitis, metabolic syndrome-like features, and tumorigenesis in murine models, supporting a microbiota-dependent inflammatory mechanism [9,16,18].

Sucralose exacerbates DSS-induced colitis through activation of the TLR5–MyD88–NF-κB axis and may contribute to colitis-associated carcinogenesis [9,24].

Nanoparticulate additives activate inflammasomes, while micro- and nanoplastics have been shown to alter immune cell populations and cytokine profiles in preclinical models [26,34,35].

Complementing mechanistic findings, large prospective cohorts such as the NutriNet-Santé study report associations between higher emulsifier consumption—including carrageenans (E407), guar gum (E412), and xanthan gum (E415)—and increased risk of T2D, providing epidemiological support for additive-related metabolic dysregulation [36].

Taken together, structural barrier disruption, metabolic reprogramming, and immune activation operate as a self-reinforcing triad linking additive-rich dietary environments to chronic inflammatory and metabolic disease.

Collectively, these perturbations reduce ecosystem resilience—defined as the capacity of the gut microbiota to resist, adapt to, and recover from environmental stressors. Within ultra-processed dietary environments, chronic multi-additive exposure may therefore compromise not only microbial composition and function but also the adaptive stability of the gut ecosystem itself.

### 2.4. Multi-Additive Synergy and the Mixture Paradigm

Most mechanistic studies of additives historically tested single additives (e.g., one emulsifier or one sweetener) under controlled conditions, which may not represent real-world UPF consumption patterns where multiple additives are repeatedly co-ingested [37]. From a risk-assessment perspective, combined exposure to multiple chemicals is increasingly recognized as a relevant framework for food-related chemical mixtures [38].

EFSA highlights the need for harmonized methodologies to assess chemical mixtures and combined exposure, supporting the scientific rationale for mixture-aware approaches in food additive evaluation [39]. In this context, synergy refers to the situation where co-exposure to multiple additives produces a gut-ecosystem effect larger (or qualitatively different) than expected from the individual effects alone.

Synergy is plausible in the gut because additives can target distinct but connected layers: (i) mucus barrier structure, (ii) microbial composition and metabolism, and (iii) epithelial/immune signaling, which together determine resilience vs. dysbiosis [40].

Mice exposed to common emulsifiers such as P80 and CMC showed an increase in metabolic syndrome-like symptoms and low-grade inflammation. On top of that, the inadequate spatial barrier structure was linked to the microbial penetration of the epithelium caused by these emulsifiers.

The major additive classes, their associated microbiota alterations, and their mechanistic effects across the three-layer model are summarized in Table 1.

A randomized controlled-feeding trial in humans reported that adding CMC to an otherwise additive-free diet perturbed microbiota composition and metabolomic profiles, with microbiota encroachment observed in a subset of participants, highlighting heterogeneity in susceptibility [20].

If emulsifiers/thickeners weaken mucus structure or increase microbe–epithelium proximity, they may sensitize the host to other concurrently ingested additives by increasing exposure of epithelial and immune sensors to microbial products.

Antimicrobial preservatives can act as selective ecological pressures, inhibiting susceptible organisms and potentially reshaping community structure in ways that alter function (e.g., fermentation capacity, colonization resistance) [41].

A systematic in vivo evaluation of antimicrobial preservatives reported that preservative exposure can impact gut microbiota and host outcomes in experimental settings, supporting the idea that repeated preservative intake contributes to community restructuring. When combined with barrier-altering emulsifiers, preservative-driven selective pressure may amplify dysbiosis by reducing beneficial competitors while barrier disruption increases the inflammatory consequences of the altered community. Reviews synthesizing additive research found that multiple additive classes can influence microbial metabolic outputs and host-relevant pathways (e.g., barrier signaling and inflammation), indicating that co-exposures may converge on shared downstream mechanisms. Because different additives can alter distinct microbial processes (growth suppression, stress responses, substrate utilization), co-exposure can plausibly destabilize microbial networks and reduce resilience even if each additive alone only produces modest shifts [42].

In the NutriNet-Santé cohort analysis, investigators empirically identified food additive mixture patterns reflecting real-world co-consumption and examined their associations with incident T2D. An increased incidence of T2D was positively associated with two of the five detected combination patterns. Two of the combination patterns that were seen included sweeteners for acidity management and associated chemicals, and the other contained various textural agents such modified starches, pectin, E412, E407, and E415 [37].

A PLOS Medicine cohort paper explicitly notes the need for experimental studies to clarify underlying mechanisms, including potential synergistic/antagonistic effects within mixtures, reinforcing multi-additive synergy as a testable hypothesis rather than a rhetorical claim [37].

Public research communications summarizing the same findings report that the observed mixtures align with common UPF categories (e.g., dairy desserts/fats/sauces and artificially sweetened beverages), supporting ecological validity of mixture patterns [37].

Multi-additive synergy is a biologically plausible and increasingly evidence-motivated paradigm because UPF diets generate chronic co-exposures and because the gut ecosystem integrates additive effects across barrier structure, microbial ecology, and host signaling. Regulatory-science frameworks already recognize the need to assess combined chemical exposures, supporting the translation of mixture thinking into food additive research and safety assessment [39].

**Figure 2:**
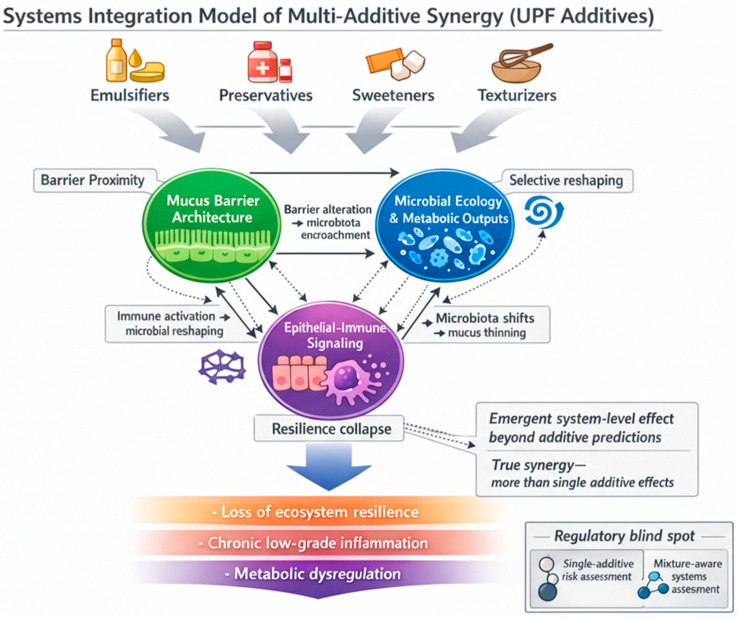
Conceptual framework for multi-additive gut–ecosystem interactions under ultra-processed food exposure. The schematic illustrates how chronic co-exposure to multiple additive classes (e.g., emulsifiers, preservatives, sweeteners, texturizers) may perturb three interconnected compartments of the gut ecosystem: (i) mucus barrier architecture, (ii) microbial ecology and metabolic outputs, and (iii) epithelial–immune signaling, which together regulate ecosystem resilience. Solid arrows indicate direct mechanistic interactions; dashed arrows denote secondary feedback and bidirectional regulatory loops contributing to system-level amplification. Simultaneous perturbations across compartments may generate emergent, non-linear effects not predictable from isolated single-additive exposures. The inset (“Regulatory blind spot”) refers to the potential limitation of conventional single-additive risk assessment frameworks in capturing mixture-driven, network-level interactions under real-world co-exposure conditions. This figure was generated using the FigureLabs AI platform (https://chat.figurelabs.ai) based on the authors’ original concept and instructions; the final image was reviewed and edited by the authors.

While the TLED Model conceptualizes the coordinated structural, metabolic, and immune cascade induced by ultra-processed dietary environments, real-world exposure rarely involves single compounds. Instead, multiple additive classes are chronically co-ingested within processed food matrices. To mechanistically contextualize this multi-additive exposure paradigm, we provide an additional systems-level framework (Figure 2) illustrating how simultaneous perturbations across barrier architecture, microbial ecology, and epithelial–immune signaling may generate non-linear amplification beyond single-additive effects. This figure does not represent a separate model, but rather a mechanistic extension of the TLED framework, emphasizing mixture-driven system-level interactions and potential limitations of single-additive risk assessment approaches.

## 3. Dietary Context as Modifier of Additive Effects: Protective Roles of Mediterranean, Vegetarian and Fiber-Rich Dietary Patterns on Gut Microbiota

Modern dietary patterns rich in UPFs expose the gut microbiota to multiple food additives whose long-term biological effects were historically underestimated. Emerging evidence indicates that emulsifiers, artificial sweeteners, preservatives, and synthetic dyes can alter microbial diversity, compromise mucosal integrity, reduce short-chain fatty acid (SCFA) production, and promote low-grade inflammation. However, the overall dietary context appears to significantly modify these additive-related effects.

Mediterranean diet, vegetarian diets, and fiber-rich nutritional models—may buffer or counteract additive-induced dysbiosis. We synthesize mechanistic, experimental, and clinical evidence demonstrating that plant-forward, fiber-dense dietary environments enhance microbial diversity, increase SCFA-producing taxa, and strengthen epithelial barrier function. We propose that dietary context acts as a critical modifier of additive impact, influencing whether microbial perturbations translate into metabolic and inflammatory pathology.

Modern Western dietary patterns, characterized by UPFs, refined sugars, saturated fats, and industrial additives, are strongly associated with microbial dysbiosis and metabolic dysfunction [43].

Increasingly, research suggests that not only macronutrient composition but also additive exposure contributes to microbiome disruption [44,45]. Importantly, dietary context may determine whether these perturbations persist or are mitigated. While additive exposure may promote dysbiosis, the surrounding dietary matrix—particularly fiber content, plant diversity, and polyphenol intake—can substantially modify these effects. Low-carbohydrate and high-protein diets illustrate the importance of distinguishing macronutrient distribution from food quality. When low-carbohydrate regimens preserve plant-derived fiber, microbial richness can be maintained or even increased. In contrast, extreme animal-based, low-fiber patterns favor bile-tolerant and proteolytic taxa, reduce butyrate-producing bacteria, and shift microbial metabolism toward branched-chain fatty acids and potentially deleterious metabolites.

Thus, microbial outcomes depend less on carbohydrate restriction per se and more on fiber preservation and food structural complexity. This distinction is essential when interpreting microbiota responses to dietary interventions.

### 3.1. Mediterranean Diet and Microbial Resilience

The Mediterranean diet, characterized by high intake of dietary fibre, polyphenols, and unsaturated fatty acids, is consistently associated with increased microbial richness and enrichment of short-chain fatty acid (SCFA)-producing taxa. Intervention studies demonstrate increased abundance of genera such as *Faecalibacterium*, *Roseburia*, Bifidobacterium, and *Prevotella*, alongside reductions in pro-inflammatory markers including CRP and IL-17 [46,47,48].

These effects are likely mediated by sustained provision of fermentable substrates and bioactive compounds that reinforce mucosal integrity, promote SCFA production, and support ecological stability within the gut microbiome. Rather than acting through a single nutrient, the Mediterranean diet appears to enhance microbial resilience through its structural and compositional complexity.

Studies report increased abundance of *Bacteroides*, *Bifidobacterium*, *Prevotella*, *Roseburia*, *Lactobacillus*, *Faecalibacterium*, and *Clostridium* clusters under Mediterranean adherence, alongside reduced *Proteobacteria* [47,49,50,51]. Increased *Prevotella*-to-*Bacteroides* ratios suggest enhanced fiber fermentation capacities [49,50].

Pagliai et al. demonstrated significant microbiome remodeling after a 3-month Mediterranean intervention, with increased SCFA production and shifts toward beneficial taxa [51]. Nagpal et al. (2018) demonstrated that a Mediterranean diet in non-human primates promotes a more diverse and functionally beneficial gut microbiome, enriched in SCFA-producing taxa, whereas a Western diet induces dysbiosis, reduced diversity, and a pro-inflammatory microbial profile associated with metabolic dysfunction [52]. Ghosh et al., in a 12-month multicenter study (*n* = 612), observed reductions in inflammatory markers including CRP, IL-17, and IL-2, alongside increased IL-10 [53].

Mechanistically, high fiber intake promotes butyrate production, strengthens tight junction integrity, enhances mucus synthesis, and modulates immune responses—potentially counteracting additive-induced epithelial damage.

### 3.2. Vegetarian Diets and Plant-Based Diets

Vegetarian diets exclude meat and fish and are typically rich in dietary fiber, plant proteins, and polyphenols while low in saturated fat and heme iron [54]. Cross-sectional and cohort studies demonstrate that vegetarians exhibit greater microbial richness compared to omnivores [55].

Plant-based diets consistently increase *Prevotella*, *Ruminococcus, Lactobacillus*, *Eubacterium*, and SCFA-producing *Clostridium* clusters [54,56,57,58]. Large-scale analyses (>20,000 individuals) reveal distinct microbial “signatures” associated with vegetarian and vegan patterns, characterized by enrichment of fiber-fermenting bacteria and reductions in bile-tolerant, inflammation-associated taxa such as *Bilophila wadsworthia* [59,60].

Functional metagenomic analyses indicate enhanced carbohydrate fermentation pathways in vegetarian and vegan microbiomes [57]. The higher fiber substrate availability likely buffers against additive-induced microbial depletion by promoting fermentative resilience and ecological redundancy.

### 3.3. Low-Carbohydrate Diets: The Role of Fiber Preservation

Dietary fiber is the primary substrate for SCFA-producing bacteria. Butyrate, acetate, and propionate support epithelial energy metabolism, reinforce tight-junction proteins, regulate immune tolerance, and suppress inflammatory signaling [61,62].

High-protein, low-fiber regimens reduce butyrate producers such as *Roseburia* spp. and *Eubacterium rectale* and increase proteolytic fermentation products, including branched-chain fatty acids and N-nitroso compounds [63]. Conversely, fiber-inclusive low-carbohydrate diets can preserve microbial richness and promote favorable phylum-level shifts [64].

Thus, fiber availability appears to be the key determinant in whether dietary modifications enhance or compromise microbial resilience. When “low-carb” diets become “low-plant, high-animal, low-fiber” diets, microbial diversity and butyrogenic capacity decline [60,63].

## 4. Lifestyle Amplifiers of Gut Ecosystem Disruption

Although ultra-processed dietary exposure represents the central perturbation discussed in this review, lifestyle factors such as chronic stress and circadian misalignment converge on the same structural, metabolic, and immune pathways described in the TLED Model. Rather than acting independently, these factors may amplify additive-driven barrier dysfunction, microbial reprogramming, and inflammatory activation, thereby lowering ecosystem resilience.

### 4.1. Chronic Stress as a Convergent Amplifier of Barrier and Immune Dysregulation

Chronic psychological or physiological stress disrupts gut homeostasis, primarily through sustained activation of the hypothalamic–pituitary–adrenal (HPA) axis and the sympathetic nervous system. Persistent elevation of cortisol and catecholamines alters epithelial integrity, mucosal immunity, and microbial ecology [65,66]

Experimental and in vivo studies demonstrate that chronic stress increases intestinal permeability through disruption of tight-junction proteins and altered mucosal immune signaling [67,68]. Stress exposure is associated with reduced production of antimicrobial peptides and decreased secretory IgA, weakening mucosal defense and barrier compartmentalization [69,70]. Increased permeability facilitates translocation of microbial products, including lipopolysaccharides (LPSs), into systemic circulation.

Sympathetic neurotransmitters such as norepinephrine directly enhance bacterial growth, iron acquisition, motility, and biofilm formation in opportunistic taxa including *Escherichia coli* and other *Enterobacteriaceae* [71,72]. Chronic stress is consistently associated with reduced abundance of beneficial commensals such as *Lactobacillus* and decreased overall microbial diversity, weakening colonization resistance [69,73]. Stress-induced inflammatory changes increase luminal nitrate availability, further favoring *Proteobacteria* expansion [74,75].

Stress-mediated increases in TNF-α and IL-6 promote low-grade inflammatory tone and may exacerbate metabolic endotoxemia [76,77].

Within additive-rich dietary environments, chronic stress may therefore lower the threshold for barrier destabilization and immune activation, amplifying the three-layer cascade and accelerating resilience erosion.

### 4.2. Circadian Rhythm Disruption and Oscillatory Instability of the Gut Ecosystem

The gut microbiota exhibits pronounced diurnal oscillations in composition and metabolic output, coordinated by host circadian clock genes including CLOCK, BMAL1, PER, and CRY [65,78,79,80,81,82,83,84]. Feeding timing acts as a primary synchronizing cue for microbial rhythmicity.

Circadian regulation influences bile acid synthesis, antimicrobial peptide secretion, epithelial turnover, and immune receptor expression, including rhythmic Toll-like receptor (TLR) activity [79,85,86,87,88]. Microbial metabolites reciprocally influence host clock gene expression via G-protein-coupled receptors and nuclear receptors such as REV-ERBα [79,85,86].

Circadian misalignment—due to night-shift work, irregular eating patterns, sleep deprivation, or jet lag—is associated with reduced microbial diversity, altered *Firmicutes:Bacteroidetes* ratios, increased *Proteobacteria*, increased intestinal permeability, and systemic inflammation [79,84,89,90,91,92,93,94,95]. Persistent circadian disruption has been linked to metabolic syndrome, obesity, T2D, and inflammatory bowel disease [9,79,90,96].

Within the TLED framework, circadian disruption affects the gut on multiple interconnected levels. When normal day–night rhythms are disturbed, intestinal permeability tends to increase and epithelial repair processes become less efficient, weakening the physical barrier between microbes and host tissues. At the same time, irregular feeding patterns alter nutrient flow and bile acid cycling, reshaping microbial metabolism and shifting the functional output of the gut microbiota. On the immune side, disrupted circadian control of TLR activity and inflammatory signaling lowers the threshold for immune activation, making the gut more reactive to both microbial products and dietary exposures. Together, these changes can amplify additive-driven perturbations and further erode ecosystem resilience. Thus, circadian misalignment may sensitize the gut ecosystem to additive-driven perturbation by destabilizing oscillatory equilibrium and reducing recovery capacity following dietary stress.

### 4.3. The Combined Impact of Diet and Lifestyle on Gut Ecosystem Resilience

Chronic stress and circadian disruption do not introduce independent pathogenic pathways; rather, they converge mechanistically with additive exposure on barrier integrity, microbial metabolism, and immune regulation. In the context of ultra-processed dietary environments, these lifestyle factors may act as amplifiers of structural destabilization and inflammatory activation.

This convergence supports a broader conceptualization of modern lifestyle as a coordinated perturbation field in which dietary additives, psychosocial stressors, and chronobiological disruption collectively shape gut ecosystem stability across the life course. Collectively, these dietary models demonstrate that microbial resilience is enhanced when diets provide structural complexity, fermentable substrates, and minimal additive exposure. In contrast, ultra-processed diets combine low fiber availability with multi-additive co-exposure, creating a permissive environment for mucus degradation, barrier dysfunction, and inflammatory dysbiosis.

Importantly, protective dietary patterns do not merely “increase good bacteria”; they reinforce ecosystem-level stability, functional redundancy, and host–microbe homeostasis. This resilience may buffer against inflammatory triggers and metabolic stressors, particularly during sensitive life stages such as early childhood and aging.

By integrating stress biology and chronobiology into the TLED Model, the present framework underscores that resilience is determined not only by dietary composition but also by the cumulative interaction between environmental exposures and host regulatory systems.

## 5. Life-Course Modulation of Diet-Induced Dysbiosis

The Three-Layer Model operates across the life course, but susceptibility and recovery capacity vary according to developmental stage, reflecting stage-specific microbial plasticity and host vulnerability.

**Figure 3:**
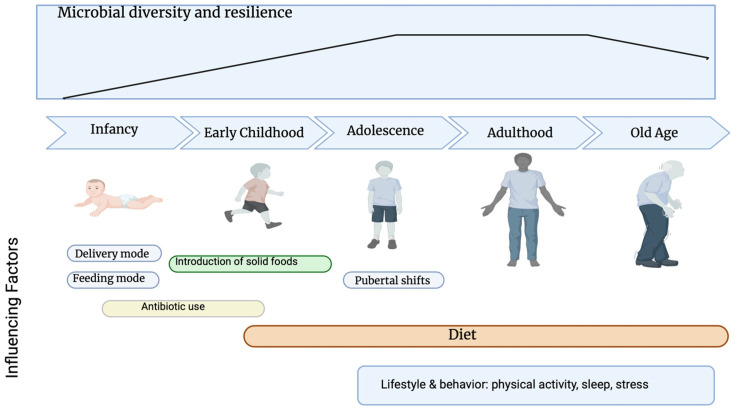
Microbial diversity and resilience across life stages. Gut microbiota diversity and ecological resilience change dynamically across the human lifespan. Microbial diversity is lowest during infancy, increases progressively throughout early childhood, and reaches relative stability during adolescence and adulthood, before declining in older age. Early-life microbiota development is primarily shaped by delivery mode, feeding practices, and antibiotic exposure, while the introduction of solid foods marks a significant transition in early childhood. Pubertal hormonal shifts contribute to microbiota remodelling during adolescence. In adulthood, the gut microbiota exhibits greater stability but remains strongly influenced by long-term dietary patterns. In older age, reduced microbial resilience is associated with age-related physiological changes and increased susceptibility to dysbiosis. Across all life stages, diet acts as a dominant modulator of gut microbiota composition, while lifestyle and behavioural factors—including physical activity, sleep, and psychosocial stress—exert continuous, cumulative effects on microbial structure and function. Created with BioRender.com (accessed on 10 January 2026).

The TLED Model operates across the life course; however, susceptibility and recovery capacity vary according to developmental stage. The gut microbiota evolves from low-diversity colonization at birth, to relative stability in adulthood, and toward reduced resilience in older age [97]. At each stage, dietary exposures interact with stage-specific microbial plasticity, immune maturation, and metabolic capacity (Figure 3).

Importantly, microbial perturbations acquired early in life may persist as latent functional imprints—what can be conceptualized as microbiological scarring—shaping microbiome responsiveness to later-life stressors, including ultra-processed dietary environments [98,99].

### 5.1. Infancy—Plasticity and Developmental Programming

At birth, the human gut is rapidly colonized by microorganisms, which vary depending on the delivery mode, antibiotic use and feeding mode (breast milk or formula).

Infancy represents a period of high microbial plasticity and tight integration between microbiota, immune education, and metabolic maturation. Delivery mode, feeding practices, and antibiotic exposure strongly influence early colonization patterns [100]. The gut microbiota of infants born via vaginal delivery is primarily colonized by maternal vaginal and fecal microbiota, dominated by genera such as *Lactobacillus* and *Prevotella* and high levels of *Bacteroides* and *Bifidobacterium*. Infants born via cesarean section have a lower microbial diversity, and the gut is colonized by microorganisms from the maternal skin and hospital environment. Key genera associated with C-sections are *Staphylococcus*, *Streptococcus*, *Corynebacterium*, and *Propionibacterium*, as well as increased *Enterococcus* and *Klebsiella* [101,102].

In addition, exclusive breastfeeding promotes a gut community dominated by *Bifidobacteria* due to the selective effect of human milk oligosaccharides (HMOs), while formula-feeding is associated with a more diverse, adult-like profile containing higher levels of *Clostridiales* and *Proteobacteria* [103].

During this window, additive-driven structural disruption (e.g., mucus thinning, altered SCFA signaling) may interfere with barrier maturation and regulatory T-cell calibration. Because foundational host–microbe interactions are being established, even transient dysbiosis may produce durable functional imprinting, persisting beyond apparent taxonomic recovery [104,105,106].

### 5.2. Childhood—Metabolic Imprinting

Although the microbiota becomes increasingly adult-like during early childhood, it remains functionally immature [107,108]. The administration of broad-spectrum antibiotics in early infancy acts as a major perturbation, causing a temporary decrease in overall microbial richness and a significant reduction in beneficial taxa like *Bifidobacterium*, with the community taking a prolonged period to normalize [109].

Within the first two years of life, the gut microbiome undergoes dramatic shifts primarily induced by the introduction of solid foods. Taxonomic similarity does not imply adult-equivalent metabolic capacity; pathways related to vitamin biosynthesis, oxidative phosphorylation, and immune modulation continue to mature through childhood and adolescence [110,111]. While some studies suggest that the gut microbiota reaches an “adult-like” configuration by 3 years, other authors support the idea that maturation continues through childhood and even adolescence [98,107,112]. For instance, children’s microbiota is shown to be enriched in *Bifidobacterium*, *Faecalibacterium*, and members of *Lachnospiraceae*, whereas adults have higher abundances of *Bacteroides*.

Disruptions during this extended developmental window—including antibiotic exposure, low-fiber intake, or early introduction of additive-rich foods—may alter microbial network consolidation and metabolic programming. Epidemiological data link early-life dysbiosis to later risk of allergic disease, obesity, metabolic dysfunction, and immune-mediated conditions. Derrien et al. [107] report that while the gut microbiota of infants (<3 years) and adults has been described, there is little data on pre-school (3–6 years), school-age (6–12 years) and adolescence (12–18 years) stages. However, research shows that the microbiome does not fully stabilize at this stage; instead, co-occurrence networks continue to consolidate, β-diversity gradually diminishes, and functional attributes continue shifting [98]. Several studies link early-life dysbiosis with increased risk for allergic diseases, asthma, obesity, metabolic disorders, autoimmunity, and even neurodevelopmental and behavioral outcomes [100,113,114,115]. The introduction of UPFs, synthetic additives (e.g., emulsifiers, artificial sweeteners), low dietary fiber, or other modern dietary patterns during complementary feeding may derail optimal microbial colonization—leading to dysfunctional interactions between the microbes and the host from early on [8,13,16,116].

Thus, the early period of life, from infancy to early childhood, represents a window of vulnerability but also an opportunity: dietary modulation (breast feeding, high intake of minimally processed, fiber-rich foods) could support healthy microbiota establishment and long-term disease prevention [106,117,118,119].

Within the TLED framework, structural and metabolic perturbations during childhood may recalibrate inflammatory and metabolic set points that persist into adulthood.

### 5.3. Adolescence—Transitional Vulnerability

Following childhood metabolic imprinting, adolescence represents a second developmental recalibration phase characterized by hormonal and immune remodeling. Metabolic shifts—including reduced SCFA production and altered bile acid signaling—may influence adiposity trajectories, insulin sensitivity, and inflammatory tone during this transitional stage. Structural and immune perturbations may interact with pubertal immune modulation, amplifying long-term cardiometabolic risk.

Adolescence also represents a period marked by physiological changes—hormonal maturation, neurodevelopment, growth and immune modulation. The interplay between diet, the microbiota, and the host during this period may therefore have unique consequences.

In adolescence, the microbiota tends towards an adult-like composition, but it continues to mature and is strongly influenced by external factors such as diet, lifestyle and environmental exposures [98].

One study showed that a 4-week lifestyle and diet intervention in adolescents positively influenced the gut microbiota, increasing the microbial diversity and the abundance of health-promoting bacterial species such as *Lachnospira*, *Alistipes*, *Barnesiella*. Functional predictions showed upregulation of genes related to energy production and metabolism [112].

A review on dietary patterns and mental health in adolescents highlights how high-sugar, low-fibre diets can lead to disruptions in the gut microbiota composition and contribute to mental health disorders such as mood disorders, anxiety, ADHD and depression via the gut–brain axis [120]. This is also supported by animal studies, which show that high-fat diets in adolescence induce long-lasting changes in the gut microbiota of rodents and expression of neuroimmune and neurotransmission-related genes in adulthood, suggesting that adolescent diets may have enduring effects on brain function via microbiota-mediated pathways [121].

In addition, adolescence may represent a vulnerable period for shaping metabolic health: diet-induced microbiota alterations during adolescence could influence energy balance, insulin sensitivity, inflammatory tone, and risk of obesity or metabolic syndrome in later life. A systematic review on microbiota–adiposity associations in children and adolescents reported inconsistent but suggestive associations between early-life microbial composition and later adiposity.

Because adolescence also represents a critical period in the development of the gut microbiome and thus general health, dietary and lifestyle interventions may still “course-correct” trajectories of dysbiosis. The improvements in microbial diversity and beneficial taxa seen after a short-term dietary change in adolescents show that these alterations can still be reversed [122].

Nevertheless, current evidence on microbiome-based interventions (prebiotics, probiotics, SCFAs) for pediatric obesity is still of low certainty. A 2025 Cochrane-style review concluded that in adolescents (10–19 years), microbiome-based interventions may lead to small reductions in BMI or waist circumference, but evidence is limited by small sample sizes, short follow-ups, and methodological heterogeneity [123].

Given the magnitude of dietary shifts many adolescents undergo (e.g., increased processed food consumption, “Western” diet patterns), more longitudinal, controlled studies are needed—especially to assess long-term metabolic, immunological, and neurobehavioral outcomes.

### 5.4. Adulthood—Cumulative Burden

In adulthood, the microbiome exhibits relative stability but remains modifiable under sustained dietary pressure. The gut microbiota generally attains a relatively stable, differentiated composition, usually dominated by the major bacterial phyla *Firmicutes*, *Bacteroidetes*, and *Actinobacteria* [97,100]. By young adulthood, the relative stability and intra-individual variation over time is modest: some studies estimate that approximately 20–30% of microbiota compositional variation is explained by intra-individual factors over time, emphasizing resilience under constant lifestyle and diet [100].

Within this stage, the Three-Layer cascade manifests as a cumulative burden: persistent barrier stress, reduced SCFA production, and chronic low-grade inflammatory activation. Long-term consumption of additive-rich, low-fiber diets is associated with depletion of SCFA-producing taxa (e.g., *Akkermansia muciniphila*, *Faecalibacterium prausnitzii*), increased permeability, and metabolic dysregulation [124].

Over decades, this “slow-burn” erosion of resilience may contribute to insulin resistance, fatty liver disease, cardiovascular risk, and inflammatory disorders [124,125].

Given the fact that adulthood often spans over decades, the cumulative effects of high intake of processed food consumption may have substantial outcomes. A recent review summarized how UPFs—defined as foods with low fiber, synthetic additives, high glycemic loads, and processing-related structural changes—are consistently associated with reduction in microbial diversity, loss of beneficial taxa, enrichment of pro-inflammatory organisms, and impairment of gut barrier function and resilience [124].

Specifically, decreased abundance of SCFA-producers such as *Akkermansia muciniphila* and *Faecalibacterium prausnitzii* is concerning: SCFAs are critical for colonocyte energy, mucin production, immune tolerance (promoting regulatory T cells), anti-inflammatory effects, and suppression of opportunistic/pathogenic bacteria [125].

Loss of these protective functions over decades explains the relationship between UPF-rich diets and chronic diseases: metabolic syndrome, obesity, T2D, non-alcoholic fatty liver disease (NAFLD), cardiovascular disease, and even colorectal cancer. Indeed, epidemiological data increasingly link high UPF consumption with such chronic disease burdens [126,127,128,129].

Moreover, chronic, low-level exposure to additives (e.g., emulsifiers compromising mucosal barrier, surfactants altering mucus layer, artificial sweeteners interfering with microbial metabolism) may produce a “slow burn” of gut barrier dysfunction, low-grade endotoxemia, immune activation, and metabolic inflammation—which are likely exacerbated by aging or other stressors [13,16].

Because the adult microbiota remains modifiable, dietary interventions, increased fibre intake, prebiotic and probiotic supplementation, and reductions in processed foods may help restore microbial balance, increase SCFA production, and improve gut barrier and metabolic health. Several authors have called for “microbiota-friendly” dietary guidelines, with a focus on reducing UPF and additive exposure [97,100,124].

Adult dysbiosis may therefore represent both the consequence of contemporary dietary exposure and the cumulative expression of earlier microbiological scarring.

### 5.5. Ageing—Reduced Resilience and Inflammaging

Ageing is associated with reduced functional redundancy, immune remodelling, and diminished recovery capacity following perturbation. Structural barrier integrity may decline due to impaired mucin production and epithelial repair since ageing is associated with alterations in microbial diversity, composition, metabolic capacity, and resilience [130,131]. Although some centenarian cohorts exhibit increased α-diversity, this likely reflects ecological heterogeneity rather than a uniform longevity signature [132]. Studies comparing elderly patients (nonagenarians, centenarians) and younger patients have reported that centenarians frequently harbour distinct gut microbiota configurations [133].

These may include higher richness/diversity, enrichment of certain beneficial or resilience-associated taxa (e.g., *Akkermansia*, *Christensenellaceae*, *Bifidobacterium*, *Lactobacillus*), and retention of SCFA-producing capacity [134,135]. A very recent study reported that centenarians had significantly higher Chao1 richness index scores compared to middle-aged adults, and that genera such as *Akkermansia*, *Lactobacillus*, and *Christensenella* were more prevalent in the centenarian group [136].

Other studies showed that taxa such as members of the phylum *Verrucomicrobia* (e.g., *Akkermansia muciniphila*)—known to support mucin integrity and gut barrier—often decrease in abundance with age, leading to decreased SCFA production, impaired mucosal protection, and increased gut leakiness [125,137].

A large-scale metagenomic/culturomic study of South Chinese centenarians documented that the relationship between age and microbiota composition is non-linear: centenarians displayed distinct configurations compared to both younger adults and elderly. For example, in centenarians, the relative abundance of *Roseburia* and *Escherichia* was increased, while several genera (e.g., *Faecalibacterium*, *Parabacteroides*, *Butyricimonas*, *Coprococcus*, *Megamonas*, *Mitsuokella*, *Sutterella*, *Akkermansia*) decreased compared with non-centenarians [135].

Moreover, longevity is associated in some populations with increased microbial richness, functional capacity for antioxidant activity, and maintenance of beneficial microbial functions—suggesting that certain microbiota configurations may support healthy aging, resilience, and possibly longevity [134,138].

Functional stability, metabolic capacity, and longitudinal resilience—not diversity metrics alone—are required to define protective microbiome configurations [139,140].

The gut community exhibits a marked reduction in key beneficial, SCFA-producing bacteria, such as *Faecalibacterium*, *Bacteroidaceae*, and *Lachnospiraceae*. This compositional decline is reflected in the reduced functional potential for broad carbohydrate metabolism and amino acid synthesis pathways [137]. Moreover, there is an increased accumulation of inflammatory commensals. These changes may contribute to gut barrier dysfunction, systemic inflammation, and aging-related morbidities [125].

In older adults, additive-driven structural and metabolic perturbations may be amplified by inflammaging and reduced colonization resistance, increasing vulnerability to frailty, metabolic disease, and inflammatory conditions [141,142]. At this stage, resilience may depend heavily on dietary quality and preservation of SCFA-producing networks [143,144].

Old age—especially in the context of processed diets, polypharmacy, reduced dietary quality, lower fiber intake, reduced physical activity, and multimorbidity—may represent a phase of heightened vulnerability, where microbiota dysbiosis contributes to frailty, metabolic, inflammatory, and neurodegenerative diseases [145,146].

However, this stage may also represent a potential window for targeted interventions (dietary, pre-/probiotic, lifestyle) aimed at maintaining microbial resilience and promoting healthy aging [147].

Age-related loss of microbial resilience may therefore unmask earlier microbiological scarring, amplifying inflammatory and metabolic consequences of diets that might have been better tolerated in earlier life [133].

Continued mechanistic and longitudinal research is essential to clarify additive-specific risks, identify vulnerable populations, and guide evidence-based dietary recommendations that protect gut microbial health [116].

## 6. Conclusions and Perspectives

Current evidence supports the concept that ultra-processed food (UPF) environments exert coordinated pressure on the gut ecosystem through three interrelated pathogenic layers: structural barrier disruption, microbial metabolic reprogramming, and sustained immune activation. Rather than acting as isolated agents, food additives operate within a multi-additive exposure paradigm that may cumulatively erode microbial resilience and promote low-grade inflammatory states linked to metabolic and chronic disease risk.

Importantly, vulnerability to additive-driven dysbiosis is not uniform across the lifespan. Early-life microbial plasticity may permit long-lasting ecological imprinting, whereas aging-associated reductions in diversity and immune adaptability may impair recovery following dietary perturbation. In adulthood, cumulative exposure appears to amplify the three-layer cascade over time, potentially contributing to metabolic and inflammatory burden.

Conversely, fiber-rich, minimally processed dietary patterns enhance ecosystem resilience by preserving short-chain fatty acid production, supporting barrier integrity, and maintaining functional redundancy within microbial communities. These findings suggest that overall dietary structure and additive burden—rather than macronutrient composition alone—are critical determinants of long-term microbiome stability.

Although much mechanistic insight derives from experimental models and human evidence remains heterogeneous, the convergence of epidemiological, translational, and mixture-based research supports the biological plausibility of additive-associated microbiota disruption. Future studies should prioritize long-term, life-course-oriented human investigations integrating additive mixture exposure assessment, repeated microbiome profiling, and clinically relevant metabolic and immune endpoints. Such approaches will be essential for translating microbiome science into actionable dietary guidance.

By formalizing additive-driven gut ecosystem perturbation within a structured, life-course-oriented systems model, this framework provides a foundation for future experimental validation, mixture-based risk assessment, and microbiota-informed dietary policy.

## Figures and Tables

**Table 1:** Major Food Additives, Associated Microbial Alterations, and Mechanistic Consequences Across the Three-Layer Model.

Additive Class	Representative Compounds	Microbial Changes (Taxa-Level)	Structural Effects	Metabolic Effects	Immune Consequences	Evidence Type
**Emulsifiers**	CMC, P80	↑ *Proteobacteria*, ↑ *Ruminococcus gnavus*, ↑ *Escherichia*–*Shigella*; ↓ *Akkermansia muciniphila*, ↓ *Faecalibacterium prausnitzii*	Mucus erosion; microbial encroachment; ↑ intestinal permeability	↓ SCFA production; altered bile acid signaling	Low-grade inflammation; colitis susceptibility; metabolic syndrome features	Murine models; controlled human feeding (CMC)
**Artificial Sweeteners**	Saccharin, Sucralose, Aspartame, Acesulfame-K	↑ *Bacteroides*, ↑ *Sutterella*, ↑ *Blautia*; ↓ *Lactobacillus*; ↓ *Clostridium* cluster XIVa	Tight-junction destabilization (claudin-1, occludin, ZO-1); oxidative stress	↓ Butyrate; altered glucose metabolism; LPS enrichment	NF-κB activation; metabolic endotoxemia; insulin resistance	Murine models; limited human data
**Colorants & Nanoparticles**	Titanium dioxide (TiO_2_), synthetic dyes	Altered *Firmicutes*/*Bacteroidetes* ratio; ↓ *Faecalibacterium* (in some models)	Epithelial stress; barrier perturbation	Limited SCFA data	Inflammasome activation; mucosal inflammation	Experimental models
**Microplastics**	PLA, polystyrene particles	↑ *Lachnospiraceae*, ↑ *Proteobacteria*; ↓ beneficial commensals	↑ Intestinal permeability	Altered microbial metabolism	Hepatotoxicity; immune activation	Murine models
**Hydrocolloids/Gums**	E407, E412, E415	Taxonomic shifts overlapping with emulsifiers	Barrier perturbation (model-dependent)	SCFA variability	Associated with increased T2D risk (epidemiology)	Prospective cohort data

## Data Availability

No new data were created or analyzed in this study.

## Abbreviations

The following abbreviations are used in this manuscript:

ADHD	Attention Deficit Hyperactivity Disorder
ADI	Acceptable Daily Intake
AI	Artificial Intelligence
BMI	Body Mass Index
BMAL1	Brain and muscle ARNT-like protein 1
CMC	Carboxymethylcellulose
CLOCK	Circadian Locomotor Output Cycles Kaput
CRP	C-reactive protein
CRY	Cryptochrome
DSS	Dextran Sulfate Sodium
E407	Carrageenan
E412	Guar gum
E415	Xanthan gum
EFSA	European Food Safety Authority
HMOs	Human milk oligosaccharides
HPA	Hypothalamic–pituitary–adrenal axis
IgA	Immunoglobulin A
IL-2	Interleukin-2
IL-6	Interleukin-6
IL-10	Interleukin-10
IL-17	Interleukin-17
LPS	Lipopolysaccharides
MAFLD	Metabolic Dysfunction-Associated Fatty Liver Disease
MyD88	Myeloid differentiation primary response 88
MUC2	Mucin 2
NAFLD	Non-alcoholic fatty liver disease
NCDs	Non-communicable diseases
NF-κB	Nuclear Factor kappa-light-chain-enhancer of activated B cells
P80	Polysorbate-80
PER	Period circadian protein
PLA	Polylactic acid
REV-ERBα	Nuclear receptor subfamily 1 group D member 1
SCFAs	Short-chain fatty acids
T2D	Type 2 diabetes
TiO_2_	Titanium dioxide
TLR	Toll-like receptor
TLR5	Toll-like receptor 5
TLED	Three-Layer Ecosystem Disruption
TNF-α	Tumor Necrosis Factor alpha
UPFs	Ultra-processed foods
ZO-1	Zonula Occludens-1
